# SyncGenie: A programmable event synchronization device for neuroscience research

**DOI:** 10.1016/j.ohx.2024.e00619

**Published:** 2024-12-24

**Authors:** Ludvik Alkhoury, Giacomo Scanavini, Petras Swissler, Sudhin A. Shah, Disha Gupta, N. Jeremy Hill

**Affiliations:** a Department of Radiology, Weill Cornell Medicine, New York, NY, 10065, USA; b Mechanical Engineering Department, New Jersey Institute of Technology, Newark, NJ, USA; c National Center for Adaptive Neurotechnologies, Stratton VA Medical Center, Albany, NY, USA; d Electrical and Computer Engineering Department, State University of New York at Albany, NY, USA

**Keywords:** Neuroscience, Event Related Potentials (ERP), Electroencephalogram, Multimodality, Stimulus synchronization and alignment, Auditory and visual stimuli, Event timing, Trigger box

## Abstract

In neuroscience, accurately correlating brain activity with stimuli and other events requires precise synchronization between neural data and event timing. To achieve this, purpose-built synchronization devices are often used to detect events. This paper introduces SyncGenie, a programmable synchronization device designed for a range of uses in neuroscience research—primarily as a “trigger box” to align neurophysiological data with physical stimulus events, among other possibilities. It can support both hardware-triggered and software-triggered pulse synchronization and can even serve as a cost-effective digitizer for real-time analysis of analog signals. We provide the complete circuit-board designs, 3D models, and Arduino code necessary to build and use SyncGenie. The board is designed for easy manufacturing and assembly, with components that can be seamlessly soldered. It includes a range of connector types required for common applications, such as 3.5 mm TRS, D-sub25, BNC, and JST-XH. Additionally, SyncGenie features a user-friendly interface that allows for experiment-specific adjustments without requiring coding expertise. Its programmability, supported by our public-domain Arduino library, provides the flexibility to adapt SyncGenie to diverse experimental protocols. Overall, SyncGenie offers enhanced functionality at a lower cost relative to commercially available trigger boxes.

## Graphical abstract

**Figure:**
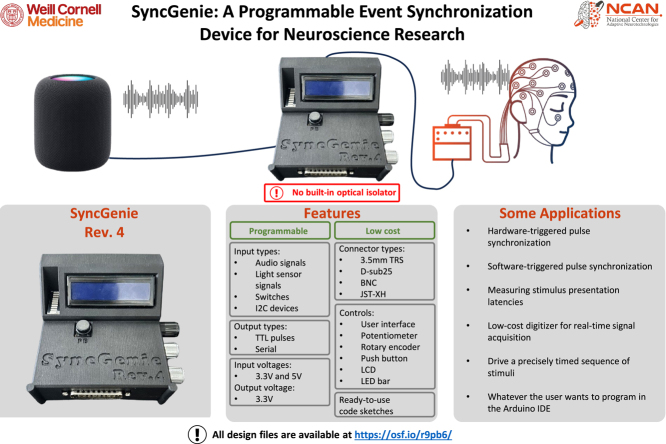

## Abbreviations used in this article

ADC: Analog-to-digital converter
BNC: Bayonet Neill–Concelman connector, frequently used for trigger lines for stimulators and other neuroscience equipment
BOM: Bill of materials
CERN OHL-P: Permissive version of CERN’s open hardware license
DAC: Digital-to-analog converter
DB25/D-sub25: A 25-pin connector from the D-subminiatures family (popularized by its use for parallel ports, and frequently used by brain signal acquisition devices for receiving trigger information)
DC: Direct current
EEG: Electroencephalography
ENIG: Electroless nickel immersion gold (metal plating process)
ERP: Event-related potential
FDM: Fused deposition modeling (3D printing process)
FR-4: Flame-retardant glass-reinforced epoxy laminate used for PCBs
GND: Ground
HASL: Hot-air solder leveling
I${}^{2}$C/IIC: Inter-integrated-circuit communication protocol
IC: Integrated circuit
IDE: Integrated development environment
JLC3DP: 3D printing service from the JLC company
JLCPCB: Printed-circuit-board manufacturing service from the JLC company
JST/JST-XH: Connector made by the JST company (the XH, specifically, has 2.5 mm pin pitch and a particular kind of plastic latch)
LCD: Liquid-crystal display
LED: Light-emitting diode
LPT: Line print terminal, a.k.a. parallel port (defines a particular pinout standard for D-sub25 connectors)
LSL: Lab Streaming Layer (popular open-source real-time synchronization library)
MJF: Multi-jet fusion (3D printing process)
RTOS: Real-time operating system
PCB: Printed circuit-board
PJRC: Name of the company that makes the Teensy development board
PTFE: Polytetrafluoroethylene, a.k.a. Teflon
PWM: Pulse-width modulation
SCL: Serial clock line (part of an I${}^{2}$C connection)
S.D.: Standard deviation
SDA: Serial data line (part of an I${}^{2}$C connection)
SLA: Stereolithography (3D printing process)
SLM: Selective laser melting (3D printing process)
SLS: Selective laser sintering (3D printing process)
SMT: Surface-mount technology
THT: Through-hole technology
TRS: Tip-ring-and-sleeve connector (a.k.a. “aux connector” or “audio jack”)
TTL: Transistor-to-transistor logic
UART: Universal asynchronous receiver/transmitter (serial port)
USB: Universal serial bus (protocol/port/connector)
V_CC_: Voltage common collector (IC power supply)

## 1 Specifications table

**Table:** 

Hardware name	SyncGenie
Subject area	•Engineering and material science
	•Electrical and biomedical engineering
	•Neuroscience
	•Educational tools and open source alternatives to existing infrastructure

Hardware type	•Programmable synchronization tool
	•Multi-trigger synchronization tool
	•Measuring physical properties and in-lab sensors

Closest commercial analog	•Wireless Trigger Hub by Wearable Sensing
	•g.TRIGBOX by g.tec Medical Engineering GmbH
	•AV Tester by Electrical Geodesics, Inc.
	•StimTrak by Brain Products GmbH

Open source license	CERN Open Hardware Licence (OHL) v2.0 Permissive

Cost of hardware	$146–$186

Source file repository	https://osf.io/r9pb6/

OSHWA certification UID	US002698

## 2 Hardware in context

Analysis of neurophysiological data in response to a stimulus often depends on knowing precisely *when* events occurred, relative to the brain activity data that are being collected [1]. Therefore, synchronization is crucial for accurately correlating neural activity with specific stimuli, responses, or events. This is especially true when the data have high temporal resolution—for example, in electroencephalography (EEG), where the phenomena under study may last small numbers of milliseconds and the temporal resolution of the recording may be as small as one millisecond.

For instance, many studies examine event-related potentials (ERPs), which are electrical brain responses detectable from the EEG record, phase- and time-locked to the presentation of a stimulus, or to a response by the research participant [2], [3]. This necessitates synchronization between the stimuli, responses, and the recorded brain signals. Typically the signal acquisition device (such as an EEG amplifier) will have a digital input port that allows events to be marked in the data-stream by sending transistor-to-transistor logic (TTL) pulses along one or more wires. The role of the “trigger box” is to detect events in the world (e.g. sound stimuli, visual stimuli, responses by the subject) and signal their occurrence by outputting these TTL pulses.

In applications that require precise synchronization, relying solely on software-based solutions (such as timestamping data files, or generating pulses in response to software commands) can be problematic. The inherent latency and jitter associated with software operations on modern multitasking computers can introduce variability, particularly when other programs are running in the background, making it difficult to achieve necessary levels of precision. A software-based system may even change its characteristics from one operating-system or driver update to the next. Problems that originate in multi-tasking delays may be mitigated somewhat by the use of a real-time operating system (RTOS) such as RTLinux, although these systems tend not to be prevalent in neuroscience laboratories, perhaps due to issues of compatibility with the wide range of equipment the field requires. Even if an RTOS is in use, in common with other software-based solutions it does not, by itself, solve the problem of detecting when a physical output actually occurred—for example, to report the output latency of the particular sound-card that is being used. Problems that originate in potential mismatches *between* timestamps may be mitigated by the use of software solutions like the popular Lab Streaming Layer (LSL) [4], [5]. LSL uses Cristian’s Algorithm to correct for clock drift between two computers, or between a computer and a particular eye-tracker or EEG amplifier or other device (assuming the device in question has its own clock and supplies timestamps for its data). Note that in both RTOS-based and LSL-based approaches, a full solution to the synchronization problem only emerges if an authoritative (likely hardware-driven) timestamp is provided, whether by a specialized sound-card or a particular model of data acquisition device. In general, one cannot assume that the input or output device has this functionality.

Whatever one’s software-based solution, its performance should be considered unknown *a-priori*, so it must be validated [6]. Validation typically uses a hardware-triggered system that delegates event detection to a single-purpose device, often called a “trigger box”. Since such a device, once obtained, serves as the gold standard during validation, the safest strategy is simply to continue using it during one’s actual experiments wherever possible, rather than reverting to a system that relies solely on software timestamps.

Various commercial trigger boxes are available to fill this role, such as the *Wireless Trigger Hub* by Wearable Sensing LLC, the *g.TRIGBOX* by g.tec Medical Engineering GmbH, the *AV Tester* by Electrical Geodesics, Inc., and the *StimTrak* by Brain Products GmbH, to name a few. In the open-source community, Bilucaglia et al. [3] have described their design for a low-cost single-channel EEG synchronization device; De Cesarei et al. [7] presented a device with a similar aim, limited to the detection of luminance changes on a screen; Aguiar et al. [8] presented another related device, geared towards rodent laboratory work. Table 1 provides a price and functionalities comparison of some commercially available trigger boxes, the ESB trigger box, and our SyncGenie (note that the price estimated for the SyncGenie assumes a minimum quantity of 5 on PCB manufacture).

The devices mentioned above either have fixed signal-processing parameters and output behavior, or at most they offer a univariate adjustment of the threshold input level that should trigger an output pulse. However, in many experimental designs, we have found it necessary to adjust additional parameters—for example, the minimum and maximum duration of the output pulse, or the refractory period that specifies the minimum time allowed between one event detection and the next. In other experiments, we have needed to customize pulse timing behavior in other ways—for example, send a trigger pulse out to a stimulator at a precise latency relative to a detected stimulus event. For these and similar tasks, over the last ten years, we have found it invaluable to use re-programmable trigger boxes based on micro-controllers.

**Table 1:**
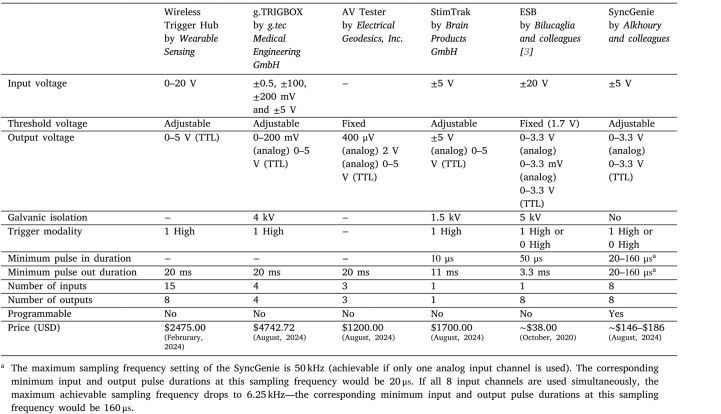
Features and price comparison between some commercially available trigger boxes, the ESB trigger box, and SyncGenie. A dash (−) indicates information was unavailable. Details about g.TRIGBOX, StimTrak, and ESB were retrieved from [3].

In this paper, we present SyncGenie, an open-source circuit-board, enclosure, and set of peripherals for the Arduino-compatible Teensy development board.^1^ When a Teensy is inserted, the SyncGenie becomes a multi-modal programmable event synchronization device that can be used for neuroscience research. SyncGenie solves the problems stated above by providing various connectors that neuroscience equipment frequently uses to communicate timing signals: D-sub25 connectors with LPT standard pinout, BNC connectors for additional digital signals, and TRS connectors for analog signals. To support more-esoteric expansions of the SyncGenie’s functionality, four pins are also reserved to allow users’ custom circuitry (including devices that use the I${}^{2}$C protocol) to be attached. Taken together, this wide range of very generic connectors makes it easy to interface the SyncGenie with a wide range of equipment and applications, not limited to EEG amplifiers but also encompassing other forms of data acquisition (fMRI, fNRIS, EMG, and others) as well as stimulators, treadmills, motion-capture systems, and anything else that uses timing pulses as input or delivers them as output (at most, some devices might require a custom-soldered connector).

We see the SyncGenie being used at three levels of user expertise:

**Table 2:** SyncGenie pinout indicating the extent of backward compatibility with different Teensy board versions.

Teensy	SyncGenie Rev. 4	Teensy Version						
Pin	Connection	4.1	4.0	3.6	3.5	3.2/3.1	3.0	LC
0	D7_OUT	PU	PU	U	U	U	U	U
1	D6_OUT	PU	PU	U	U	U	U	U
2	D5_OUT	P	P	P	P			
3	D4_OUT	P	P	P	P	P	P	P
4	D3_OUT	P	P	P	P	P	P	P
5	D2_OUT	P	P	P	P	P	P	
6	D1_OUT	P	P	P	P	P	P	P
7	D0_OUT	P	P	P	P			
8	SELECTOR_BUTTON	P	P	P	P			
9	SELECTOR_A	P	P	P	P	P	P	P
10	SELECTOR_B	P	P	P	P	P	P	P
11	BNC2	P	P	P	P			
12	BNC1	P	P	P	P			

13	D9_LED	PL	PL	L	L	L	L	L
14	TRS3_TIP	AP	AP	AP	AP	A	A	A
15	TRS3_RING	AP	AP	A	A	A	A	A
16	POT2	A	A	AP	A	A	A	AP
17	POT1	A	A	AP	A	A	A	AP
18	JST_PIN3	API	API	A I	A I	A I	A I	A I
19	JST_PIN4	API	API	A I	A I	A I	A I	A I
20	TRS2_TIP	A	A	AP	AP	AP	AP	AP
21	TRS2_RING	A	A	AP	AP	AP	AP	A
22	TRS1_TIP	AP	AP	AP	AP	AP	AP	AP
23	TRS1_RING	AP	AP	AP	AP	AP	AP	AP

24	TRS4_TIP	AP	–			–	–	–
25	TRS4_RING	AP	–			–	–	–
26	LCD_RS	A	–			–	–	–
27	LCD_EN	A	–			–	–	–
28	LCD_D4	P	–			–	–	–
29	LCD_D5	P	–	P	P	–	–	–
30	LCD_D6		–	P	P	–	–	–
31	LCD_D7		–	A	A	–	–	–
32	D8_LED		–	A	A	–	–	–

33	BUTTON	P	–	A	A	–	–	–
34	D0_IN		–	A	A	–	–	–
35	D1_IN		–	AP	AP	–	–	–
36	D2_IN	P	–	AP	AP	–	–	–
37	D3_IN	P	–	AP	AP	–	–	–
38	D4_IN	A	–	AP	AP	–	–	–
39	D5_IN	A	–	A	A	–	–	–
40	D6_IN	A	–	A D${}^{*}$	A D${}^{*}$	–	–	–
41	D7_IN	A	–	A D${}^{†}$	A D${}^{†}$	–	–	–

Key	(Relevant Pin Capabilities/Notes)
A	ADC (analog input)—supports analogRead()
P	PWM (pulse-width-modulated pseudo-analog output)—supports analogWrite()
D	DAC (true analog output)—supports analogWrite()
U	UART interface 1—supports Serial1.begin() etc. (pin 0 receives, pin 1 sends)
I	I${}^{2}$C interface 0—supports Wire.begin() etc. (pin 18 is SDA, pin 19 is SCL)
L	Onboard LED
–	Pin not available—short-footprint Teensy models have no access to TRS4, the input (male) D-sub25, the LCD screen, the top-side push-button, and the (spare) second LED from the top on the bar-graph array.
*	The Teensy 3.6 and 3.5 diagrams do not indicate a pin 40 on the front. The pin that is addressable from firmware as 40 is actually on the back. Instead, this physical position is marked “A21/DAC0” with no pin number. The board library headers define A21 as 66, so this is the number to use.
†	The Teensy 3.6 and 3.5 diagrams do not indicate a pin 41 on the front. The pin that is addressable from firmware as 41 is actually on the back. Instead, this physical position is marked “A22/DAC1” with no pin number. The board library headers define A22 as 67, so this is the number to use.

1. Expert: the SyncGenie can be seen as no more than a breakout board for the Teensy, enabling access to the above-mentioned connectors for whatever firmware the user might care to program using the Arduino IDE. Our library headers associate pin numbers with components (first two columns of Table 2), and the rest can be up to the user.
2. Intermediate: We provide a number of libraries for the Arduino IDE—most crucially the SyncGenie library [9]. This library provides high-level classes with names like Trigger, Switch, Wheel and Gauge, that wrap up common event-detection and de-bouncing logic efficiently, and in a way that users can adapt easily by manipulating a few parameters. The library also provides a framework that simplifies the construction of user menus to be displayed on the SyncGenie’s LCD screen, as well as utilities for saving settings to (and loading them back from) persistent storage. Our separate Keyhole library [10] also provides a simple framework that allows the SyncGenie (or other Arduino-compatible device) to receive commands and variable values efficiently from a computer via its USB serial connection.
3. Basic: We provide a ready-made firmware sketch, FourJacksMenuDriven.ino, which allows the SyncGenie to be used as a versatile, configurable, standalone trigger-box without the user having to do any programming at all (or even connect the SyncGenie to a computer). This firmware supports up to 8 independently-processed input channels, allowing each one to be configured to detect onsets of oscillatory signals (e.g. audio waveforms), onsets of DC signals (e.g. light-sensor readings), or simple switch closures (e.g. for subjects’ button-press responses). Via the SyncGenie’s on-screen menu, the user can configure each channel’s signal detection threshold, the minimum duration of its output pulse, the hold time of the output pulse (i.e. the maximum gap in the input through which the output pulse will be sustained), the refractory period (i.e. the minimum gap between one output pulse and the next), and the pin on which the output pulse is delivered. Full details are provided in the dedicated user manual [11].

Given the SyncGenie’s flexibility and reprogrammability, it can be adapted to many purposes in a neuroscience laboratory. We see its principal use-cases as follows, but the list is by no means exhaustive:

1. The main use-case of the SyncGenie, and the one we implement for basic-level users, is what we have elsewhere [1] termed “hardware-triggered pulse synchronization”, i.e. the rapid detection of physical events and generation of corresponding output pulses. This is the same use-case as the other commercial and open-source products mentioned above, but with greater flexibility of parameterization.
2. The SyncGenie can also be used for software-triggered pulse synchronization—for example, when a command needs to be issued directly by a computer to trigger a stimulator or similar device. When employing it this way in our own experiments, we usually use the SyncGenie in conjunction with BCI2000, an open-source software platform for real-time biosignal acquisition, processing and feedback [12], [13]. The BCI2000 source distribution [14] contains a plug-in called SerialInterface, complete with a matching example sketch leveraging the Keyhole library [10]: together, these allow microcontrollers to respond to real-time software contingencies in a manner that can be flexibly configured.
3. It can be used to combine software-triggered and hardware-triggered pulse synchronization, e.g. for the purpose of measuring audio or video latencies (as exemplified by our previously-published study of audio latencies [1]).
4. It can also be used as a low-cost digitizer for real-time signal acquisition, which can be a useful and flexible tool when prototyping and diagnosing experimental setups. Again, the BCI2000 software provides a useful complement: its source distribution includes, in the SerialWidgetADC submodule, two example sketches that make use of the SignalAcquisition [15] and Keyhole [10] libraries for the Arduino IDE. One sketch acquires analog signals (for example, sound via the TRS jacks). The other sketch acquires signals from an MPU6050 accelerometer/gyroscope device via the Teensy’s I${}^{2}$C connection, which the SyncGenie breaks out on its four-pin JST-XH connector. (The MPU6050 and similar devices offer a potential platform for performing hardware-triggered pulse synchronization for, and analysis of, vibrotactile stimuli.)
5. It can be used to drive a precisely-timed sequence of stimuli, without the need for a computer. (For example, in ongoing unpublished studies, we are using it this way to trigger vibrotactile stimuli in customizable sequences.)

The timing precision of the SyncGenie in any of these applications cannot, of course, be guaranteed in “intermediate” or “expert” mode: performance will only be as good as the code the user writes, and any new application or code change must be tested empirically before the user can be confident of its behavior. For “basic”-mode hardware-trigger synchronization, provided the user does not increase the sampling rate beyond the maximum indicated by the built-in diagnostic, our results reported in Section 8 should provide an accurate guide to expected performance.

## 3 Hardware description

Our repository at https://osf.io/r9pb6/ provides the following:

• raw and editable schematics and PCB sketches used to create the SyncGenie Rev. 4 PCB layouts: our originals in .json format for EasyEDA [16], as well as versions converted to .kicad_pcb format for KiCAD [17];
• the 3D model of the enclosure and the LCD holder, in .step format suitable for FreeCAD [18] and other software packages;
• the exported manufacturing files: Gerber, pick-and-place and BOM for the PCBs, and .stl files for the 3D model of the enclosure and the LCD holder.

**Fig. 1:**
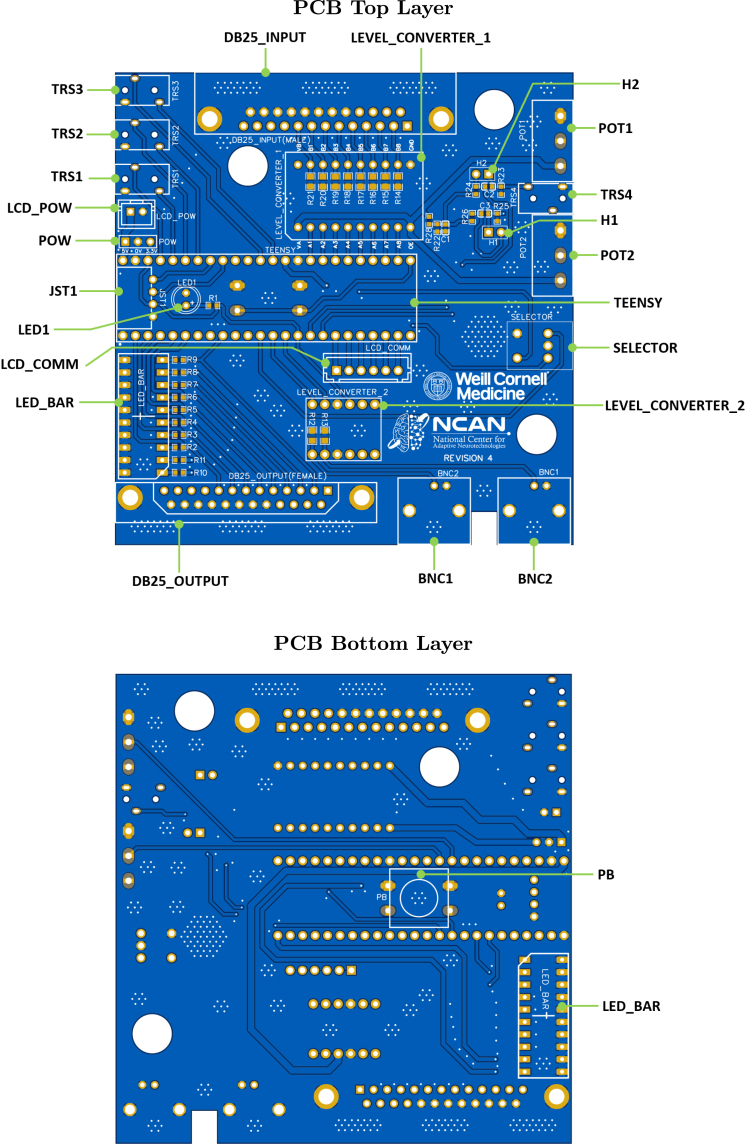
The top and bottom views of the main SyncGenie PCB are shown in the upper and lower images, respectively.

**Fig. 2:**
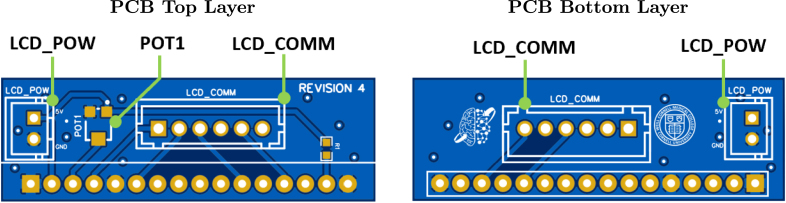
The top and bottom views of the auxiliary PCB are shown in the left and right images, respectively.

**Fig. 3:**
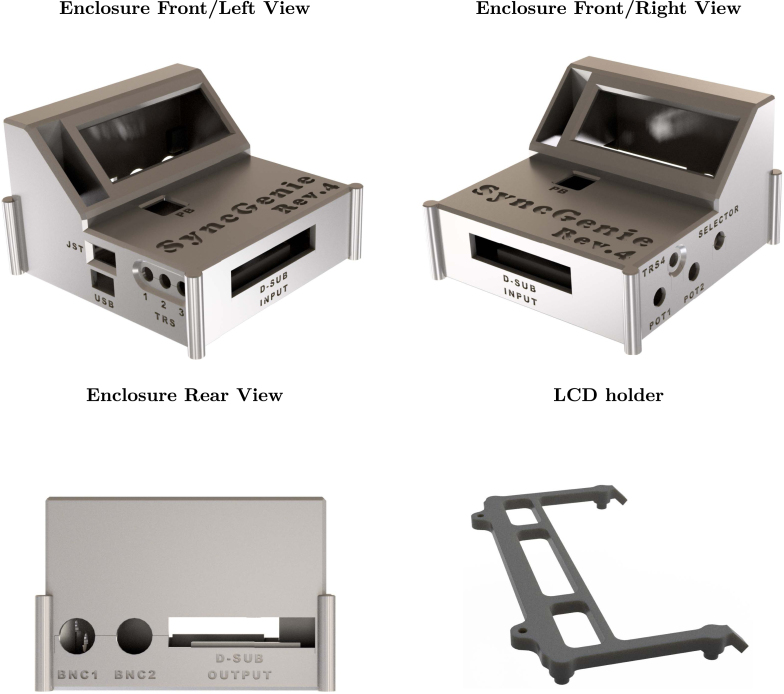
3D model of the SyncGenie enclosure from multiple views and the LCD holder (bottom right).

Fig. 1 shows the top and bottom layers of the main PCB, and Fig. 2 similarly shows the auxiliary PCB. In Fig. 3 we show the 3D model of the enclosure as well as the LCD holder. The major blocks and components embedded in the boards are discussed in the following subsections.

### 3.1 Teensy development board

The central component in our design is the PJRC Teensy development board, labeled TEENSY. The SyncGenie board can support short-footprint Teensies (such as Teensy 3.2 and 4.0) as well as a long-footprint Teensies (such as Teensy 3.6 and 4.1). Short Teensies have fewer pins than longer models, although in a compatible arrangement—as a result, some functions will work as intended when a short Teensy is plugged in, and some will be unavailable. Table 2 summarizes the SyncGenie’s pinout when used with various Teensy models. Beyond short-footprint Teensy models’ inability to use a subset of the components, and differences in processor speed, there are few functional differences between versions (differences between the versions’ pin capabilities lie mostly in which pins can or cannot perform pulse-width modulation, which we anticipate to be needed rarely if at all in SyncGenie’s use-cases). Generally, to unlock the SyncGenie’s full potential, we recommend the use of a long model—preferably the Teensy 4.1 [19].

The Teensy is located in the center of the PCB with its micro-USB port at the left edge (Fig. 1, left image). When users develop their own microcontroller code and cabling configurations to create custom solutions, there is always some risk of shorting electronic components by mistake and damaging them. In common with all the vulnerable electronic components of the SyncGenie, the Teensy has header pins soldered along its long edges (many vendors will supply the Teensy pre-prepared in this way) which clip solderlessly into spring connectors on the board. This design makes a damaged Teensy (or level converter, LCD screen or LED bar) very easy to replace in the SyncGenie.^2^

### 3.2 D-sub25 connectors

The SyncGenie board contains two D-sub25 connectors: a female connector labeled DB25_OUTPUT located in the bottom left of the PCB that is used to output signals, and a male connector labeled DB25_INPUT located in the top center (Fig. 1, upper image) of the PCB that is used for input signals. Both female and male connectors follow the parallel port configuration: pins 18 through 25 are connected to the ground, and pins 2 through 9 are employed as data bits 0 through 7, respectively. The data pins of DB25_OUTPUT are soldered to eight LEDs on the LED bar (labeled LED_BAR and located on the bottom left of the PCB). This offers a visual indicator every time a pulse is output on one of the parallel-port data pins. Moreover, the male DB25_INPUT connector is connected to an 8-channel logic level bi-directional converter module TXS0108E (labeled LEVEL_CONVERTER_1 on the PCB). This level converter is intended to protect the Teensy from damage in use-cases where it must receive pulses from other devices that use 5 V rather than 3.3 V as logic HIGH.

### 3.3 BNC connectors

The board contains two female BNC connectors, labeled BNC1 and BNC2, that can be used as digital inputs or outputs. The connectors are located on the bottom right of the PCB (Fig. 1, upper image). They are connected to two channels of a 4-channel bi-directional logic level converter module (the KeeYees 4-channel logic level converter, labeled LEVEL_CONVERTER_2 on the PCB). This level converter is intended to protect the Teensy from damage in use-cases where it must receive pulses from other devices that use 5 V rather than 3.3 V as logic HIGH.

### 3.4 TRS connectors

We equipped the board with a total of four female 3.5 mm tip-ring-sleeve (TRS) connectors, labeled TRS1 through TRS4. The first three are located on the top left side of the PCB (Fig. 1, upper image) and their tips and rings connect directly to the respective pins of the Teensy board (see the pinout in Table 2). Note that portions of the signal that go below 0 V are not captured by the Teensy’s analog-to-digital converters, which therefore create a similar effect to a half-wave rectifier. The tip and ring of these connectors can be configured as inputs, as outputs, or as a combination of both. It is sometimes useful to output digital-HIGH voltage on one of the contacts (we usually use the tip) and read from the other: we would do this, for example, when using a ‘nurse call’ cord as a push-button for collecting research participants’ responses, since these devices typically connect tip to ring when the button is pressed; we would also configure things this way when using a light sensor to detect visual stimulus onsets (an output pin in the HIGH state suffices to power many suitable phototransistors). For auditory stimuli, the half-wave-rectified signals of TRS1 through TRS3 usually suffice to support adequate onset detection and triggering. However, if the full wave is needed for any reason, users have the option of using TRS4, whose ring and tip are each connected to their own DC bias circuit that shifts the signal up by V_CC_/2 = 1.65 V. This bias allows us to measure the negative-going parts of the original signal as positive voltages smaller than the bias. In this configuration, the ring and tip of TRS4 can only be used as analog inputs; however, we have added two open headers labeled H1 and H2 (to the left of TRS4 on the circuit-board): shorting these will disable the bias circuits and allow TRS4 to function similarly to the rest of the TRS connectors.

### 3.5 JST connector

The board also contains a male four-pin JST-XH connector (2.5 mm spacing) located in the middle left of the board and labeled JST1 (Fig. 1, upper image). The pins are connected to ground, V_CC_, and two additional pins that can be used either generally for arbitrary digital input/output purposes, or specifically as the SDA/SCL pair of the Teensy’s default I${}^{2}$C interface, enabling communication with other devices that use the I${}^{2}$C data transfer protocol.

### 3.6 Power headers

The SyncGenie board also contains a three-pin header (labeled POW) connected to 5 V, 3.3 V, and GND. This header can be used to power sensors or devices when testing them with the SyncGenie and it provides a power node in case needed. A power LED (labeled LED1) lights up when the SyncGenie is powered.

### 3.7 User control components

Additionally, the board contains two potentiometers labeled POT1 and POT2 which are located at the top right of the PCB (Fig. 1, upper image). The potentiometers are connected to analog-capable input pins and can hence be used for parameter adjustment. A simple normally-open push-button, labeled PB, is mounted on the reverse side of the main PCB (which faces upwards when enclosed, so the button is on the top surface of the finished device, as seen on the front/left and front/right views of Fig. 3). Lastly, a rotatory encoder, incorporating a push-button of its own, is available on the right center side of the PCB and is labeled SELECTOR (seen on the front/right view of Fig. 3). These user-interface controls can be coded for arbitrary purposes in the microcontroller firmware. In our default firmware, the potentiometers and top-side push-button are unused, whereas the encoder is used to navigate the configuration menu (which allows users to adjust settings to achieve a reasonably wide range of functionality without the need to do any Arduino coding themselves).

### 3.8 LCD connectors and auxiliary board

The SyncGenie incorporates a 16 × 2 character LCD, which is mounted via the small auxiliary PCB shown in Fig. 2. The main PCB and auxiliary PCB each have two headers that allow them to be connected: the headers labeled LCD_COMM on both boards provide a data connection via 6-pin JST-XH connectors, and the headers labeled LCD_POW on both boards provide power to the LCD via 2-pin JST-XH connectors. The auxiliary PCB also contains a potentiometer labeled POT1 that allows the brightness of the LCD backlight to be adjusted.

## 4 Design files summary

**Table:** 

Design filename	File type	License	Location of the file
Main & auxiliary PCBs	raw schematics, *.svg; PCB sketches, *.kicad_pcb; EasyEDA source, *.json; manufacturing files, *.grb & *.csv	CERN OHL-P 2.0	https://osf.io/r9pb6/

3D designs	source, *.step; manufacturing, *.stl	CERN OHL-P 2.0	https://osf.io/r9pb6/

BOM of other components	*.ods	CERN OHL-P 2.0	https://osf.io/r9pb6/

Firmware code	Arduino IDE source, *.ino	CC0 (Public Domain)	https://arduino.cc/reference/en/libraries/syncgenie/

• Main and auxiliary PCBs: we provide design files of SyncGenie’s PCBs. That is the raw and editable EasyEDA files (.json format) as well as KiCAD PCB files (.kicad_pcb format). We also provided manufacturing files that can be used directly to produce the PCBs. These are Gerber (.grb), pick and place (.csv), and BOM (.csv) files.
• 3D designs: we provide raw editable 3D design files (in .step format suitable for FreeCAD [18]) for the SyncGenie’s enclosure as well as the internal LCD holder. We also share the corresponding .stl files that can be used directly in slicing software to generate a 3D print.
• BOM of other components: we provide a BOM that contains all the remaining components we used to build the SyncGenie.
• Firmware code: we developed an Arduino library with examples that can be directly updated and run on the SyncGenie. Additionally, we created a ready-made firmware sketch, FourJacksMenuDriven.ino, which allows the SyncGenie to be used as a versatile, configurable, stand-alone trigger-box without the user having to do any programming at all.

## 5 Bill of materials (BOM)

Given the BOM’s extensive length, we have included a detailed and comprehensive BOM in the project’s main repository https://osf.io/r9pb6/, listing all electronic components along with their part numbers, manufacturers, and supplier IDs. We report the price in USD as provided on the supplier’s website on August 13th, 2024.

We compute the cost of making the SyncGenie as $146–$186 per unit, depending on the chosen quality options for PCB manufacturing and 3D printing. This per-unit cost assumes a minimum quantity of 5, reflecting the minimum quantity required by the PCB manufacturing company. If one were definitely only interested in making *one* SyncGenie, the PCB company’s minimum batch size would mean that some costs would be wasted: one would need to buy around $400 of materials and services, of which around $170 would be wasted (i.e. not used in making the first SyncGenie, and only useful for making more SyncGenies); about $55 worth of generic materials would also be left unused, but would likely be reusable in other projects.

## 6 Build instructions

We provide step-by-step instructions on how to manufacture and build the SyncGenie. Building SyncGenie requires very basic soldering knowledge for through-hole and surface-mounted components.

### 6.1 PCB manufacturing

As previously mentioned, we provide all the files needed in the PCB manufacturing process. These files are Gerber, Pick-and-Place, and BOM and they can be found in the repository, https://osf.io/r9pb6/. This directory contains two PCB models; the main SyncGenie board and the auxiliary board. We have used JLCPCB (https://jlcpcb.com/)^3^ to manufacture the boards since it provides a wide variety of base materials and colors and other PCB specifications. We used the highest PCB specification quality for the main SyncGenie board PCB. We have used polytetrafluoroethylene (PTFE) as the base material. We picked a PCB thickness of 1.52 mm with an electroless nickel immersion gold (ENIG) surface finish with a gold layer of 2 µ” (1 µ” = 0.0254 µm). We also chose the copper weight on the top and bottom to be 1 oz/ft${}^{2}$ (∼35 µm). We used two ground planes (top and bottom) with a total of 435 via connectors. The minimum track width was 0.5 mm and the maximum was 1.25 mm.

For the auxiliary PCB, we used a standard quality PCB with flame retardant (FR-4) as the base material. We picked a PCB thickness of 2 mm with a hot air solder leveling (HASL) (with lead) surface finish. We also chose the copper weight on the top and bottom to be 2 oz/ft${}^{2}$. We used two ground planes (top and bottom) with a total of 9 via connectors. The track width was 0.5mm.

It is worth mentioning that the price of PCB is significantly influenced by the specifications and materials used for production. We provide in Table 3 a price comparison between different design specifications and materials as well as their advantages and disadvantages [20], [21], [22].

**Table 3:** Price comparison between different design specifications and material offered by JLCPCB. We have shown the prices for the manufacturer’s minimum order of 5 PCBs, as at August 8th, 2024.

Base material	PCB specifications	Price (5 boards)	Advantage	Disadvantage
FR-4	PCB thickness of 2 mm HASL (with lead) surface finish, 2 oz/ft${}^{2}$ of copper on the top and bottom layers.	Main PCB: $52.60^a^ Aux. PCB: $47.20^b^	Low-effective, easy to fabricate, safely used in various environments	Limited use in high-frequency applications, Less suitable for applications that generate significant heat

PTFE	PCB thickness of 1.52 mm. ENIG surface finish of 2 µ ” gold layer. 1 oz/ft${}^{2}$ of copper on the top and bottom layers	Main PCB: $80.37^a^ Aux. PCB: $78.59^b^	Ideal for high-frequency applications, operates at temperatures higher than FR-4, durable in harsh environments	High cost, difficult to fabricate, long production lead time

a Add $43.57 for purchase and soldering of 5 main boards’ SMT components (27 resistors + 3 capacitors per board).

b Add $39.82 for purchase and soldering of 5 auxiliary boards’ SMT components (1 resistor + 1 potentiometer per board).

### 6.2 3D printing

We provide a 3D model (.stl files) for an enclosure designed to fit the PCBs. We also provide a holder model that we use to easily mount the LCD onto the enclosure.

For 3D printing, we have used JLC3DP to 3D print the enclosure. The enclosure was built using the Multi Jet Fusion (MJF) technology that uses PA12-HP Nylon. This method generated a high resolution and fine features on the print. JLC3DP provides other less-expensive alternatives to MJF printing, which could result in a lower-quality product. We provide in Table 4 a price comparison between different technologies and materials offered by JLC3DP along with their advantages and disadvantages [23], [24], [25], [26], [27].

**Table 4:** Price comparison of different 3D printing technologies and materials offered by JLC3DP. Here we compare the price to 3D print the top and bottom pieces of the SyncGenie enclosure and the LCD holder. The price was obtained on August 8th, 2024.

Technology	Materials	Price	Advantage	Disadvantage
Stereolithography (SLA)	Ledo 6060 Resin	$14.42	High resolution, smooth surface finishes	Parts generally brittle, post-curing required

Fused Deposition Modeling (FDM)	PLA plastic	$16.46	Very low cost, wide range of materials	Lower resolution and accuracy compared to other methods, visible layer lines, anisotropic mechanical properties.

Selective Laser Sintering (SLS)	3201 PA-F Nylon	$33.32	No need for support structures, good mechanical properties, high accuracy	Rough surface finish, post-processing needed, higher equipment costs

Multi Jet Fusion (MJF)	PA12-HP Nylon	$50.43	Excellent mechanical properties, faster build times than SLS, good surface finish	Limited material options, expensive equipment, post-processing required

Selective Laser Melting (SLM)	316L stainless steel	$215.50	High density complex parts, excellent mechanical properties	High cost, extensive post-processing, potential for internal stresses and distortion

### 6.3 Component soldering

**Fig. 4:**
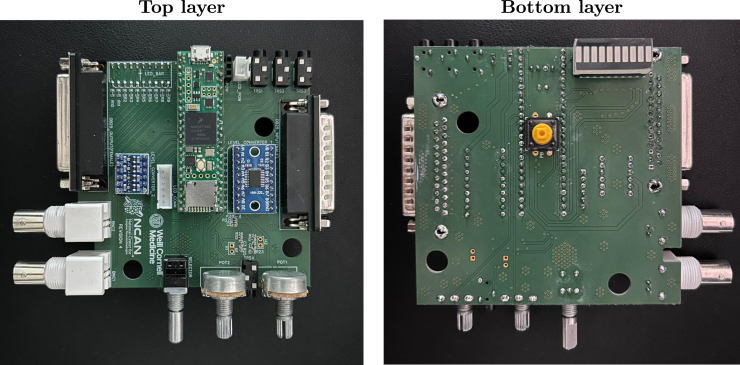
SyncGenie main PCB after all components were soldered.

**Fig. 5:**
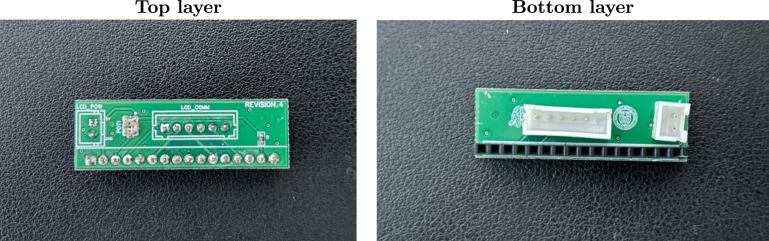
Auxiliary PCB after all components were soldered.

After the PCBs are manufactured, the electronic components should be soldered onto the boards. Our design contains two types of components; through-hole technology (THT) and surface mount technology (SMT). SMT components are typically smaller which allows for smaller size PCBs. However, their small size poses more difficulties when assembling and soldering these components onto the boards. Fortunately, many PCB manufacturers provide soldering services for SMT components. For our design, we used the mounting and soldering service provided by JLCPCB (which comes at a cost that we provide as a footnote in Table 3) to solder all resistors and capacitors on the main board, as well as the resistor and potentiometer on the auxiliary board. All the rest of the THT components were manually soldered at our lab. The Teensy, the level converters, and the LED bar were not soldered directly onto the board. Instead, we soldered female headers where the Teensy and the level converters can be plugged in, and integrated circuit (IC) base sockets where we plug in the LED bar. This allows us to swap modules easily and replace modules in case they burn out. Fig. 4, Fig. 5 show the SyncGenie’s main PCB and auxiliary PCB, respectively, after all components are soldered.

### 6.4 Assembly

**Fig. 6:**
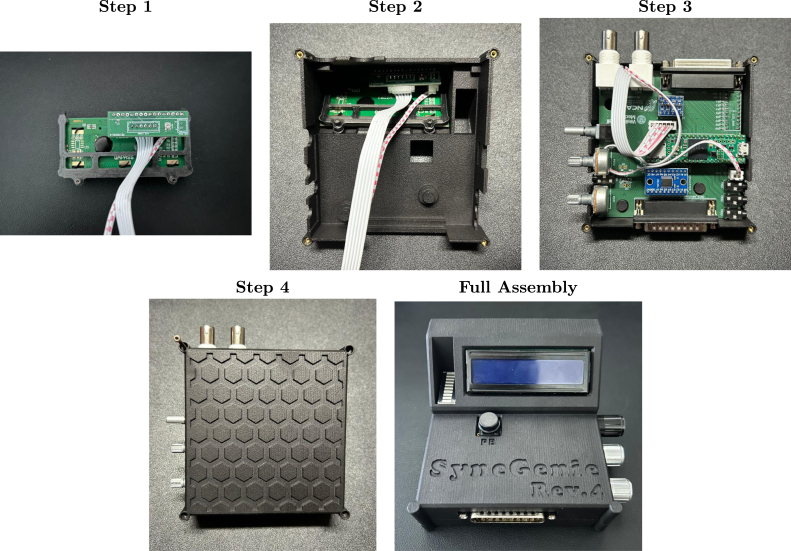
Step-by-step illustration on how to build the SyncGenie following the steps described in Section 6.4. The last panel shows the fully assembled device.

Once all components are soldered onto the boards and the enclosure is 3D printed, the last step is to assemble all the SyncGenie components following the steps below (illustrated in Fig. 6):

1. Plug the power and communication cables into the auxiliary PCB, then place the LCD holder onto the LCD board and plug the LCD board and auxiliary PCB together (Fig. 6, Step 1).
2. Flip the top piece of the enclosure and place the LCD assembly inside of it. Use two M2 × 5 screws to hold it in place (Fig. 6, Step 2).
3. Place the SyncGenie main PCB into the enclosure. Pass the power and communication cables of the LCD through the gap between the BNC connectors. Connect the power and communication cables to the main SyncGenie PCB (Fig. 6, Step 3).
4. Connect the bottom piece of the enclosure to the top piece. Use four M2 × 15 screws to secure the corners (Fig. 6, Step 4). Lastly, connect the washers and nuts that come with the BNC connectors, the potentiometers, and the selector to ensure that these components are mounted securely. Add the potentiometer and selector knobs if desired.

The last panel of Fig. 6 (labeled ‘Full Assembly’) shows the fully assembled SyncGenie.

### 6.5 Programming

The SyncGenie Arduino library can be installed via the Arduino IDE’s Library Manager window. (We also recommend installing the Keyhole library, which is not a dependency of SyncGenie because the library itself does not use it, but some of the example sketches require both.) The SyncGenie library includes various example sketches for learning and testing parts of the library: once the SyncGenie library is installed, these sketches can be accessed by launching Arduino IDE, then going to **File** → **Examples** → **SyncGenie**.

One of the “example” sketches, FourJackMenuDriven.ino, is intended less for teaching the user how to program the SyncGenie, but rather as a complete firmware implementation that allows the SyncGenie to be used as a user-configurable standalone trigger box. This program leverages the LCD screen and selector knob to provide a user interface, so the SyncGenie does not need to interface with a computer at run time, even if the user wants to change parameters and save them. The interface allows the user to configure SyncGenie’s TRS connectors as various types of trigger or switch input, to adjust their parameters, to redirected their output pulses to selected D-sub25 pins and/or BNCs, to perform diagnostic measurements, and to access on-screen documentation. More details can be found in the paper’s repository (https://osf.io/r9pb6/).

**Fig. 7:**
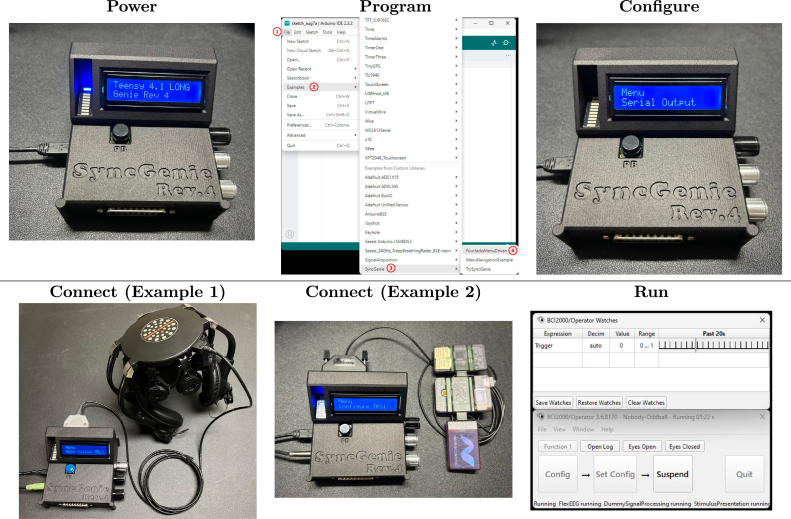
Examples of SyncGenie’s operation with the DSI7 headset by wearable sensing (Example 1) or the FlexEEG by NeuroCONCISE (Example 2).

## 7 Operation instructions

### 7.1 User instructions

When using the SyncGenie, five steps should be followed; **Power**, **Program**, **Configure**, **Connect**, and **Run**. We illustrate these steps in Fig. 7 using an auditory oddball experiment run using the BCI2000 software [12] in conjunction with a Wearable Sensing DSI7 headset and a NeuroCONCISE FlexEEG^TM^. The oddball experiment consists of abrupt, frequent standard and rarer deviant stimuli presented in rapid, randomized sequences. The stimuli were square-wave beeps of 340 ms duration with a fundamental frequency of either 400 Hz (standard) or 575 Hz (deviant), as well as a variety of novel deviant sounds. The stimuli were presented in random order in a 1-second repeating rhythm [28]. The presented stimulus was delivered via an audio splitter, simultaneously to the participant’s headphone’s and to the SyncGenie’s TRS1 port. We used the tip of TRS1 as the input channel. To connect to the DSI7 (Fig. 7, lower left), we used the D-sub25 to LX40-12P cable supplied by the manufacturer, and relied on the headset’s built-in optical isolation. By contrast, the FlexEEG (Fig. 7, lower middle) is unusual in that it receives triggers via a serial connection instead of raw TTL pulses, but the SyncGenie can support this on pin 8 of its rear D-sub25 connector. The manufacturer built for us a serial cable with a D-sub25 connector at one end and a three-pin connector at the other. An optical isolator was integrated into this cable, since the FlexEEG does not implement this safety feature internally (see Section 7.2). The steps, illustrated in Fig. 7, were as follows:

1. Power: Connect SyncGenie to the computer via a USB cable. This step is shown in the top left panel of Fig. 7. When the SyncGenie is powered on, the LCD will activate and display a message, and an indicator LED will flicker. Additionally, the power LED will illuminate, with the light visible through the USB connector.
2. Program: For first-time use (or if the firmware needs to be changed), open the appropriate firmware sketch in the Arduino IDE, select “Teensy 4.1” from the board menu, and press the “Upload” button. In this demonstration we used our general-purpose default firmware, FourJacksMenuDriven.ino, for which a more comprehensive user manual is also available [11].
3. Configure: Identify and activate the proper input and output connectors based on the experimental protocol. For this demonstration, we used the SyncGenie’s on-screen menu to disable processing of all inputs except the tip of TRS1, following the instructions in Sections 4–5 of the user manual [11]. We then set the tip’s threshold to 50, and its refractory period to 800 ms, to reflect the characteristics of the auditory stimuli we were using. To accommodate the FlexEEG’s serial communication protocol, we also turned on “Serial Output” mode (for the DSI7 and most other devices, we ensure this option is turned off). We then saved the settings.
4. Connect: Establish a physical connection using the proper cables between the sensor and the SyncGenie. For this example, the purpose-built optically-isolating serial cable is shown in the lower middle panel of Fig. 7). Other sensor hardware will use its own type of cable (typically supplied with the equipment, and often ending in a D-sub25, as in the lower left panel). Note also the TRS cable plugged into the SyncGenie’s TRS1 port on the left side.
5. Run: Launch the experiment software and ensure that the system works as designed. The lower right panel of Fig. 7 shows the train of trigger signals sent by the SyncGenie, collected and incorporated into the datastream by the FlexEEG, and received and displayed by BCI2000.

### 7.2 Safety considerations

Hardware used for recording biological signals from humans must be constructed according to certain safety standards that prevent macro shocks in the event of electrical faults and accidental misuse [29]. For example, an EEG headset or amplifier that has wired power, data, or trigger connections must isolate these connections. For signal data and triggers, this usually means optical isolation.

The SyncGenie, in common with other trigger boxes — and also in common with most ordinary computer equipment — belongs firmly on the “machine” side of this isolation barrier and not on the “human” side. As such, the SyncGenie does not implement any safety isolation itself. The user *MUST* ensure that there is adequate optical isolation between the SyncGenie and any circuitry that directly contacts a human subject (e.g. sensors that measure biosignals).

In most cases, the necessary isolation will be implemented in the acquisition device. The user’s main responsibility, then, is to verify that this is indeed the case, by consulting the device’s user manual or by contacting the manufacturer. For example, in our laboratories, we mostly use Dry Sensor Interface headsets by Wearable Sensing and wired amplifiers by g.tec, all of which implement onboard optical isolation for their trigger inputs and other wired connections. We also use the g.Nautilus by g.tec—a headset that communicates wirelessly with, and is therefore isolated by an air gap from, the base station that receives trigger information. When used correctly with these devices, the SyncGenie (and other custom trigger circuitry) present no known or anticipated safety hazard to the person being measured. Note that “used correctly” means that trigger circuitry should only be connected to an acquisition device’s purpose-built digital-input port—it should never be connected to analog ports that are intended for biosignal sensors, references, or grounds.

By contrast, some particularly compact or low-cost acquisition devices may lack onboard optical isolation. For example, to use the SyncGenie with the FlexEEG^TM^ headset by NeuroCONCISE, it was necessary to develop a custom cable that incorporated an optical isolator into its D-sub25 connector. Similarly, to use the SyncGenie with an OpenBCI Cyton board or other low-cost device, one would also have to build optical isolation into the circuit, as per the example/instructions provided on the OpenBCI website [30]. This approach should be taken when using any hardware that cannot be positively verified as having adequate optical isolation.

In addition to optical isolation, the risk of shock can be further mitigated by powering the SyncGenie (and any other laboratory devices that allow it) over USB from a laptop running on battery, disconnected from the mains power supply. This usually ensures that only DC potentials are present in the laboratory setup, and at a low overall maximum voltage.

Finally we should note that, as with most other open-source hardware offerings, we cannot provide any safety guarantees for the SyncGenie, whether the user follows the above guidelines or not. Note in particular that the SyncGenie has not been tested, certified, or approved for use in clinical applications. In all applications, it is constructed and used at the user’s own risk. The authors disclaim all liability to the extent permitted by applicable law.

## 8 Validation and characterization

As we mentioned in Section 2, the SyncGenie can be used as a development platform in “expert” or “intermediate” mode, in which case its performance will only be as good as the user’s programming. It is very easy to violate inadvertently the real-time conditions required to ensure good timing performance. Therefore, the user’s programs (or changes to existing programs) must always be carefully and conservatively validated before they are used in real experiments, and we cannot make any projection of the results.

We can, however, report representative performance of the SyncGenie in “basic” mode. We developed a versatile firmware program called FourJacksMenuDriven.ino. This program provides a menu-driven interface in which a user can use the selector knob and LCD screen to configure TRS1 through TRS4 to detect audio onsets (triggering from an oscillatory signal), to detect visual onsets (triggering from a DC signal from a light sensor), or to register button-presses or other switch closures (for example, from a “nurse call” cord or similar TRS push-button). To support this, as well as users’ other custom firmware solutions, we prepared an Arduino library called SyncGenie which provides, among other things, a Trigger class. Each Trigger instance samples one analog-capable input pin and monitors for changes in its oscillatory or DC signal, and outputs a pulse on up to two digital output pins to mark the change onset. The class provides parameterization of:

• sampling frequency,
• triggering threshold,
• minimum pulse duration—i.e. the minimum time between the initial threshold-crossing and termination of the output pulse,
• pulse increment duration—i.e. the minimum time between the *most recent* threshold-crossing and termination of the output pulse (this governs pulse duration—if set to zero, the output pulse is of fixed duration; if greater than zero, the pulse will tend to be sustained as long as the sound input signal continues);
• refractory period—i.e. the minimum time between one output-pulse onset and the next (this is for de-bouncing the trigger output).

The principal performance measure of interest will be the delay in detecting the onset of an incoming signal and outputting the pulse. This delay should not only be short relative to the EEG phenomena to be studied, but also invariant across repetitions under uniform conditions. Event-related estimation of mid-latency EEG potentials has been shown to deteriorate if the delay has a standard deviation of more than 10 ms [31]. From our own experience, and to encompass shorter-latency analyses, we would recommend a stricter tolerance: specifically, the total delay should stay consistently below 1–2 ms. This outcome will be the main focus of our validation, in Section 8.1. As subsidiary outcome measures, we will also report on output pulse duration (Section 8.2) and power consumption (Section 8.3).

Wishing to test the limits of this firmware’s performance, we measured it under the most-challenging conditions: all eight channels (tip and ring of each TRS) were configured as independent triggers, each with a separate associated Trigger instance, rather than as simple switches (which would be less computationally demanding). Tests were performed with a Teensy 4.1 at its standard clock speed of 600 MHz.

### 8.1 Response timing performance

On each loop() (the fundamental cycle of operation of an Arduino sketch) the firmware checks whether it is time for a sample to be gathered. If the firmware cannot keep up with processing demands, the answer will always be yes, whereas if it can perform all its operations in time, there will tend to be “idle” loops which return without sampling. The firmware includes a menu-accessible idle-loop counter for diagnostic purposes, as well as an option for varying all channels’ sampling rates together. We found that, with all eight channels running as Triggers, 6.25 kHz (160 µs per sample) was the maximum sampling rate at which idle loops still remained. This, then, is the default sampling rate in our firmware. Reducing the number of Trigger instances can increase this bound—for example, with six triggers and one switch, this increases to 10 kHz, i.e. 100 µs per sample; with only one trigger, it is possible to run at 50 kHz (20 µs).

**Fig. 8:**
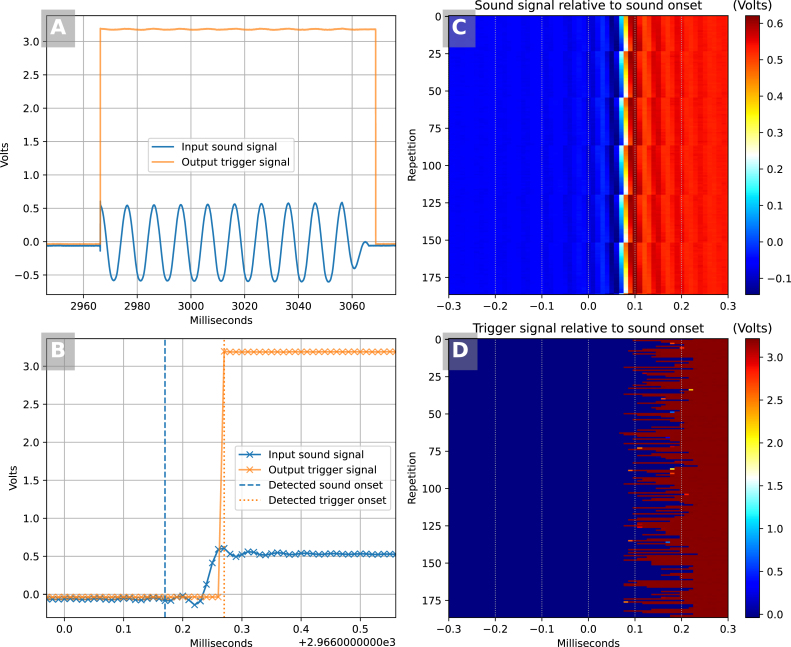
Input–output latency and jitter for a single channel that detects onsets of an auditory signal. Panel A: one full stimulus together with the resulting trigger output signal from the SyncGenie, digitized at 100 kHz. Panel B: the same stimulus and trigger output, zoomed in to show the sub-millisecond interval either side of the sound and trigger onsets, as computed by our analysis routines and plotted as vertical dashed and dotted lines, respectively. Panel C: raster-plot of the 187 repetitions of the sound signal with time zero aligned to the time at which our analysis routine detects sound onset. Panel D: raster-plot of the 187 trigger onsets relative to the same time zero as in panel C (their latency is 0.157 ms ±0.048ms S.D.).

In this configuration, running at 6.25 kHz, we were interested to characterize the latency between the onset of an abrupt sound signal fed into the SyncGenie and the onset of the output pulse by which the SyncGenie signals that the onset has been detected. We generated the sound signal shown in blue in Panel A of Fig. 8: an abrupt-onset (cosine-phase) sinusoid at 100 Hz, tapering away to silence after 100 ms. We played this, digitized at the standard audio rate of 44.1 kHz, through our PC’s soundcard and split the analog signal so that it went both to the SyncGenie’s first trigger channel and to a National Instruments PCIe6321 digitizer. The SyncGenie’s corresponding trigger output was also fed into the digitizer, where both input and output signals were digitized together at 100 kHz. We played 187 repetitions of the sound with random uniform inter-stimulus intervals between 0.25 s and 1 s.

We detected sound onsets in the recorded data using custom Python code. Our code first detected the time at which the absolute sample-to-sample change in voltage exceeded a certain threshold, and then group-corrected these onsets (shifting each onset estimate until its associated time-locked epoch had maximum correlation with the mean epoch) to avoid the onset estimates being unduly influenced by small random peri-threshold differences in sample voltage. An example illustrating onset detection is shown in Panel B, zoomed-in to a fraction of a millisecond; the aggregated results of sound onset detection are shown in the raster plot of Panel C. Based on these input onset estimates, the output onsets are then shown as a raster plot in Panel D. The input–output latency was estimated to be 157 µs±48 S.D. (min = 70, max = 240) across 187 repetitions.

The mean latency (about one sample, at the rate we used) must be considered a somewhat fungible estimate because it depends on an arbitrary choice of threshold value (looking at Panel B, it is possible to argue that the sound “really” began tens of microseconds either before or after the blue dashed line, depending on your criterion). However, the jitter estimate of ±48  µs is invariant to this consideration.

We did not implement any sample-and-hold circuitry, which means that the triggers operated strictly sequentially on the input, and different channels might therefore measure slightly different voltages even if connected to the same source. When fed the same input signal, the output pulses of the first and eighth (last) trigger channel were measured to be about 117 µs apart ±6 S.D., reflecting the fact that our library code takes about 16–17 µs to sample and process each sample for each channel. (On some trials, the sound signal actually crossed the detection threshold *between* the time at which first trigger sampled the signal and when the eighth sampled it; the delay therefore allowed the eighth channel to pick up the sound onset one whole sample *before* the first—so in these cases we observed a difference of −43 µs instead of +117 µs.)

Taken together, the overall effects of discrete sampling, sequential trigger processing and jitter led to perturbations that were still clearly confined to the sub-millisecond scale. This level of precision is more than adequate for most purposes in non-invasive neuroscience (for example, in our experience of the analysis of event-related potentials in EEG, jitter in the low single-digit milliseconds makes no appreciable difference to the results).

For more-challenging applications where even greater precision is necessary (for example, the analysis of brain-stem evoked potentials whose components are 1ms apart), we can expect improvements if we reduce the number of channels, thereby allowing higher sampling rates, shorter overall latencies, and a smaller maximum channel-to-channel discrepancy. In the limit: after using the on-screen menu to reduce the number of active channels to 1, and then to increase the sampling frequency to the new maximum of 50 kHz that this now allowed, we measured input–output latencies of 94 µs ±8 S.D. Barring future improvements in the library code and potential gains from overclocking the Teensy, this is the limit of the precision we expect from the SyncGenie in detecting onsets of oscillatory signals.

### 8.2 Pulse duration

To characterize the SyncGenie’s precision in generating pulses of a prescribed length, we played 200 independently-generated 25 ms bursts of random noise from a computer sound card, with random inter-burst intervals between 0.25 s and 1 s. The audio output was connected to the SyncGenie’s TRS1 while it was monitoring all 8 input channels at 6250Hz. The TTL output pulses were fed into a digitizer and acquired at 100 kHz, as described above. When configured to produce pulses with a fixed pulse duration of at least 10 ms, the SyncGenie’s pulses were found to be 10.083 ms ±0.005 S.D. (all 200 measurement were either 10.080 or 10.090, suggesting that the precision was limited by our measuring device rather than the SyncGenie).

To characterize the SyncGenie’s ability to produce pulses at least as long as the input stimuli, we re-ran the test after configuring a minimum output duration of 10 ms and a duration *increment* of 10 ms—the increment represents the maximum length of gap in the stimulus that the SyncGenie will tolerate before dropping the output voltage to zero. In this condition, the pulses were measured at 34.765 ms ±0.326 S.D.This is consistent with the 25 ms stimulus duration, the added tolerance of 10 ms, and the random timing of the last supra-threshold sample in the different noise bursts.

### 8.3 Power consumption

We measured the power consumption of the SyncGenie using an Eversame USB 3.0 Tester. We tested the SyncGenie under normal usage conditions, i.e. with the LCD screen active, the default FourJacksMenuDriven.ino firmware running, and the LEDs consequently responding to the TRS input signals. By varying the trigger channel thresholds, we could choose to measure with each of the trigger-output LEDs either off or on. Depending on the number of LEDs lit, we obtained readings that varied from 600 mW to 750 mW. Holding the number of LEDs constant, the standard deviation was around 10 mW across repeated measurements. Power consumption was not noticeably affected by the configured sampling rate: variation in sampling rate may shift the Teensy from predominantly performing analog reads and floating-point operations, to predominantly busy-waiting in so-called “idle” loops, but in either case there are no sleep operations as such, and the clock speed of the processor itself remains constant.

The Teensy’s internal temperature sensor readings were found to rise from around 40 °C to around 50 °C over the first hour, and then remain stable over a subsequent hour of continuous operation. (This was also not noticeably affected by the sampling-rate configuration.)

### 8.4 Example EEG data

To illustrate the SyncGenie’s operation in an EEG experiment, we recorded data at 300Hz from one adult participant, using the Wearable Sensing DSI24 headset and the BCI2000 software platform [12], [13]. The paradigm was an auditory “oddball” in which the participant listened to sequence of discrete sounds presented every 1 s. Most of these stimuli (270 out of 410) were “standard” 340 ms beeps at 400Hz; these were randomly interspersed with a minority (80 out of 410) of “target” 575Hz beeps (to which the participant was asked to respond using a button), and a smaller minority (60) of “distractor” sounds that each only occurred once and required no response. This is a version of the classic P3a/P3b paradigm described by Polich [32]—the particular software implementation and stimuli used here are identical to those described in one of our previous studies [28].

**Fig. 9:**
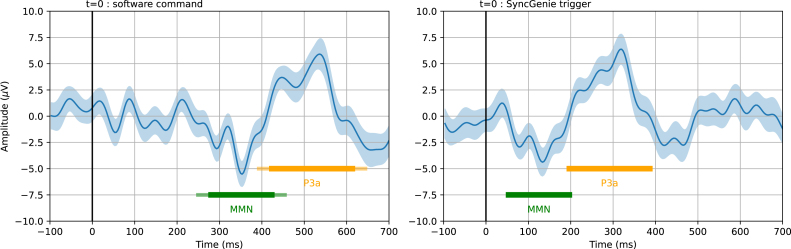
Averaged event-related-potential differences in an auditory “oddball” EEG experiment. Both panels show a time-locked average (± one standard error) of the difference between the EEG response to “distractor” sounds vs. “standard” tones, measured at electrode Pz. Left and right panels show analyses of the same data, averaged differently: in the left panel, time zero is defined as the time at which the command to play the audio was issued by the software; in the right panel, time zero is defined as the time at which the rising edge of the SyncGenie TTL pulse was recorded in the data-stream following detection of the physical stimulus. The green bar marks the identifiable mismatch negativity (MMN) component, a negative peak in the difference wave that is expected 100–250 ms after stimulus onset. The orange bar marks the P3a component, a positive peak expected at around 300 ms. In the right panel, these components occur at the expected latencies. In the left panel, they appear later due to the audio stimulus latency (which was 227 ms on average according to the TTL pulse timing) and the bars have been given fuzzy edges to represent the associated jitter of ±30.7ms. Note also the differences in peak morphology, due to the jitter.

The results are shown in Fig. 9. Our analysis of the timing of the TTL pulses relative to the software timestamps revealed that we had used a computer/sound-card/sound library configuration with a long audio latency (227 ms on average) and a high associated jitter (±30.7ms S.D.). This jitter had a sufficiently large effect on the time-locked averaging that it appreciably changed the shape of the expected negative peak (mismatch negativity, or MMN) and positive peak (P3a) in the distractor-standard difference wave. Since audio latencies may vary from a few milliseconds to a few hundred milliseconds, depending on many interacting factors in one’s hardware and software [1], this illustrates the need for a trigger device such as the SyncGenie—at the very least to validate the system’s performance, and preferably to run the actual experiments. The SyncGenie was configured to deliver trigger pulses of fixed 10 ms duration; the pulses themselves did not perturb the EEG signal (if they had, signal artifacts would be noticeable at time 0–10 ms in the right panel of Fig. 9).

## CRediT authorship contribution statement

**Ludvik Alkhoury:** Writing – original draft, Validation, Methodology, Investigation, Data curation, Conceptualization. **Giacomo Scanavini:** Visualization, Resources. **Petras Swissler:** Visualization, Resources. **Sudhin A. Shah:** Visualization, Resources, Funding acquisition. **Disha Gupta:** Visualization, Resources, Funding acquisition, Conceptualization. **N. Jeremy Hill:** Writing – review & editing, Writing – original draft, Validation, Supervision, Software, Methodology, Investigation, Funding acquisition, Data curation, Conceptualization.

## Ethics statement

EEG data (Section 8.4) were collected under protocol number 1584762 approved by the institutional review board of the Stratton VA Medical Center. Informed consent was obtained from the participant.

## Declaration of competing interest

The authors declare that they have no known competing financial interests or personal relationships that could have appeared to influence the work reported in this paper.

## Footnotes

1 The current SyncGenie revision is compatible with multiple Teensy models but is optimized for the version 4.1. The Teensy is not an ideal component of an open-source project because it is not, itself, fully open-source. However, thus far we have stuck with the Teensy due to a combination of features that we have found otherwise hard to match—especially at its price point, and especially earlier in the SyncGenie’s development history. The most important features are the high signal-to-noise ratio of its analog-to-digital conversion, its high processor speed and large RAM, and its simplicity of setup for custom programming (by neuroscientists who might not have deep experience in embedded-systems development) via the Arduino IDE. Open-source alternatives may now provide competitive performance, which we have not yet fully explored.

2 The risk of accidental mis-configuration also highlights the safety requirement that trigger box inputs and outputs must be optically isolated from the any devices that contact human research participants. This should be implemented by the biosignal acquisition hardware itself—if not, an optical isolator must be interposed (see notes on safety in Section 7.2).

3 Other PCB manufacturers offer comparable services—for example, DKRed (https://www.digikey.com/en/resources/dkred), OSH Park (https://oshpark.com/), and PCB Way (https://www.pcbway.com/), to name a few..
